# Markers of neutrophil activation and neutrophil extracellular traps in diagnosing patients with acute venous thromboembolism: A feasibility study based on two VTE cohorts

**DOI:** 10.1371/journal.pone.0270865

**Published:** 2022-07-28

**Authors:** Philip Smith, Axel Rosell, Maria Farm, Maria Bruzelius, Katherina Aguilera Gatica, Nigel Mackman, Jacob Odeberg, Charlotte Thålin

**Affiliations:** 1 Department of Medicine, Karolinska Institutet, Solna, Stockholm, Sweden; 2 Theme of Emergency and Reparative Medicine, Karolinska University Hospital, Stockholm, Sweden; 3 Division of Internal Medicine, Department of Clinical Sciences, Danderyd Hospital, Karolinska Institutet, Stockholm, Sweden; 4 Department of Molecular Medicine and Surgery, Karolinska Institutet, Stockholm, Sweden; 5 Department of Clinical Chemistry, Karolinska University Hospital, Stockholm, Sweden; 6 Coagulation Unit, Department of Hematology, Karolinska University Hospital, Stockholm, Sweden; 7 UNC Blood Research Center, Division of Hematology, Department of Medicine, University of North Carolina at Chapel Hill, Chapel Hill, North Carolina, United States of America; 8 Department of Protein Science, Science for Life Laboratory, KTH-Royal Institute of Technology, Solna, Sweden; 9 Department of Clinical Medicine, UiT The Arctic University of Norway, Tromsø, Norway; 10 Division of Internal Medicine, University Hospital of North Norway (UNN), Tromsø, Norway; Ludwig-Maximilians-Universitat Munchen, GERMANY

## Abstract

**Background:**

Venous thromboembolism (VTE) diagnosis would greatly benefit from the identification of novel biomarkers to complement D-dimer, a marker limited by low specificity. Neutrophil extracellular traps (NETs) have been shown to promote thrombosis and could hypothetically be used for diagnosis of acute VTE.

**Objectives:**

To assess the levels of specific markers of neutrophil activation and NETs and compare their diagnostic accuracy to D-dimer.

**Methods:**

We measured plasma levels of neutrophil activation marker neutrophil elastase (NE), the NET marker nucleosomal citrullinated histone H3 (H3Cit-DNA) and cell-free DNA in patients (n = 294) with suspected VTE (pulmonary embolism and deep vein thrombosis) as well as healthy controls (n = 30). A total of 112 VTE positive and 182 VTE negative patients from two prospective cohort studies were included.

**Results:**

Higher levels of H3Cit-DNA and NE, but not cell-free DNA, were associated with VTE. Area under receiver operating curves (AUC) were 0.90 and 0.93 for D-dimer, 0.65 and 0.68 for NE and 0.60 and 0.67 for H3Cit-DNA in the respective cohorts. Adding NE and H3Cit-DNA to a D-dimer based risk model did not improve AUC.

**Conclusions:**

Our study demonstrates the presence of neutrophil activation and NET formation in VTE using specific markers. However, the addition of NE or H3Cit-DNA to D-dimer did not improve the discrimination compared to D-dimer alone. This study provides information on the feasibility of using markers of NETs as diagnostic tools in acute VTE. Based on our findings, we believe the potential of these markers are limited in this setting.

## Introduction

Venous thromboembolism (VTE), comprising deep vein thrombosis (DVT) and pulmonary embolism (PE), is a common and potentially fatal condition [1]. An extensive list of predisposing factors and a clinical presentation often mimicking other diseases provides for a diagnostic challenge. The clinical approach in the outpatient setting has essentially remained unchanged for almost two decades. First, the clinical or pre-test probability (PTP) of VTE is assessed using clinical decision rules (CDR), e.g. Wells score or Geneva score [2–4]. Second, VTE is ruled out in low probability cases with a normal D-dimer test. Finally, VTE is confirmed or dismissed in high-probability cases through diagnostic imaging [5, 6].

The strength of D-dimer as a VTE biomarker lies in its high sensitivity and negative predictive value [7]. However, elevated D-dimer levels are not only seen in thrombosis but also in cancer, pregnancy, inflammation, infection, trauma [8] and with increasing age [9]. Despite the introduction of age-adjusted cut-offs [10], its specificity for VTE is at best around 70% with a false positive rate of around 25–30% [11]. This limitation, in combination with Wells score being applicable only to the outpatient setting, brings considerable draw-backs to the current diagnostic strategy including an over-utilization of computer tomography pulmonary angiography (CTPA) [12].

A novel biomarker-based confirmatory test would thereby provide a long sought-after complement in VTE diagnostics and has therefore been the objective of extensive research. Several candidates have been proposed: soluble P-selectin, factor VIII, microparticles (extracellular vesicles), different adhesion molecules (ICAM-1, VCAM-1), C-reactive protein, protein C and S, fibrinogen and cytokines (IL-6, IL-10) [7]. None of these biomarkers have, however, met requirements for clinical implementation.

Neutrophils can externalize decondensed chromatin as a response to strong stimuli in a process referred to as neutrophil extracellular trap (NET) formation [13]. Although first described in response to microbial pathogens, several stimuli have been shown to induce NET formation including soluble P-selectin [14], interleukin-8 [15], and granulocyte-colony stimulating factor [16] and NETs have been implicated in a variety of non-infectious disease settings. In vitro studies have demonstrated that NETs have many different prothrombotic effects on the intravascular micro-environment. This includes platelet adhesion, activation and aggregation as well as promoting thrombin formation [17]. Numerous animal studies have established a role of NET formation in thrombotic disorders [16–18], and NET components have been found in human venous and arterial thrombi [19, 20]. Markers of NET formation have thereby been proposed novel candidate markers in VTE diagnostics [17, 19, 21–23].

Being core components of the mesh-like NET structures, increased circulating levels of cell-free DNA (cfDNA), granular proteins, e.g. neutrophil elastase (NE) and myeloperoxidase (MPO) and nucleosomes have often been used as markers of NET formation. Diaz et al. demonstrated an association between increased levels of plasma DNA, plasma MPO and DVT [21]. Similarly, increased levels of circulating nucleosomes and neutrophil elastase-α1-antitrypsin complexes were observed in DVT patients in a study by van Montfoort et al. [23]. However, the interpretation of these markers as NET markers has been strongly questioned as these markers do not solely reflect NET formation but also general tissue damage and neutrophil activation [24, 25]. Prior to NET release, the enzyme peptidylarginine deiminase 4 citrullinates histone H3 in the nucleus contributing to chromatin decondensation, hence citrullinated histone H3 (H3Cit) is regarded a NET-specific marker [26]. Nonetheless, the association between H3Cit and acute VTE has not been studied.

The aim of the study was to investigate the potential of markers of neutrophil activation and NETs as diagnostic biomarkers in VTE, compared to and in combination with D-dimer. We therefore measured levels of nucleosomal H3Cit (H3Cit-DNA), in addition to cfDNA and NE, in two clinical cohorts of patients with confirmed symptomatic VTE and patients with a clinical suspicion of VTE but in whom VTE was ruled out as well as in healthy controls.

## Materials and methods

### Study participants

**Fig 1:**
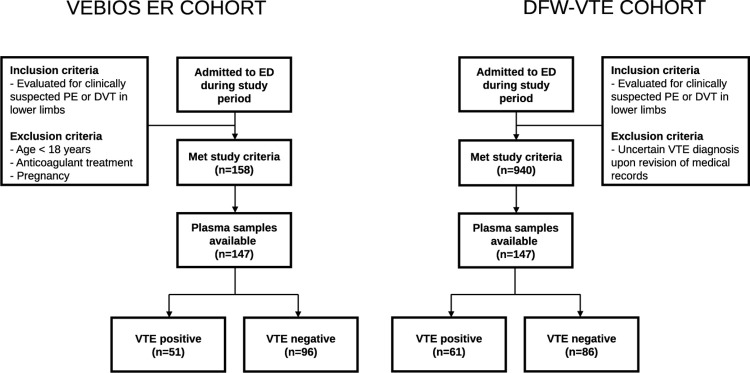
Flow chart describing inclusion of study participants.

Study participants were recruited from two different VTE cohorts, VEBIOS ER and DFW-VTE (Fig 1). In short, all patients were included based on clinical suspicion of VTE (PE and/or DVT in a lower limb). VTE was objectively confirmed using diagnostic imaging and ruled out either by diagnostic imaging or a negative D-dimer test in combination with Wells score in accordance with current guidelines. All blood samples were collected upon admission to the emergency room (ER), before a VTE diagnosis was determined and before any administration of anticoagulant treatment. Time from symptom onset to ER admission varied between the study participants, from hours to days. Similarly, there was some variability regarding the time to blood draw based on individual patient triage level but in general samples were collected within a few hours from admission. All participants with available plasma samples were included from both cohorts.

### VEBIOS ER

VEnous thromboembolism BIOmarker Study (VEBIOS) has been previously described by Bruzelius et al. [27]. As part of this prospective cohort study, a subgroup of patients was included at the ER at Karolinska University Hospital in Solna, Sweden, between December 2010 to September 2013. All patients were over 18 years of age at time of inclusion. DVT was confirmed using ultrasonography and PE using CTPA. A total of 158 patients (52 VTE positive) were included. Blood samples were collected in EDTA and 0.109 mol/L (3.2%) sodiumcitrate vacutainer tubes by direct venipuncture and sent within 30 minutes to the Karolinska University Laboratory for further processing. After centrifugation at 3000 × g for 10 minutes, plasma aliquots were snap frozen and stored at -80°C. Data was collected from questionnaires filled out by the attending doctor at the ER as well as extracted from electronic medical records (EMR). The extractor was blinded to levels of H3Cit-DNA, NE and cfDNA. The study protocol was approved by the regional Ethical Review Agency in Stockholm, Sweden (Dnr 2010/636-31/4). Informed written consent was obtained from all study participants in accordance with the Declaration of Helsinki.

### DFW-VTE

DFW-VTE (D-dimer, Fibrin monomer and Wells score in VTE study) is a prospective single-center study and has previously been described by Farm et al. [11]. All patients were included at admittance to the ER of Karolinska University Hospital in Huddinge, Sweden, between April 2014 and May 2015. DVT was confirmed by either doppler ultrasonography or CUS whereas PE was confirmed by either CTPA or ventilation/perfusion lung scintigraphy. A total of 954 patients (125 VTE positive) were included. In a subset of 174 patients, plasma samples were stored for secondary analyses. Out of these, plasma samples were available from 147 patients (61 VTE positive) for the current study. All relevant data was extracted from EMR. The extractor was blinded to levels of H3Cit-DNA, NE and cfDNA. Blood was collected in 0.109 mol/L (3.2%) sodiumcitrate vacutainer tubes by direct venipuncture. After centrifugation at 3000 × *g* for 10 minutes the samples were frozen at -80°C within one hour. The samples were thawed once in 37°C water-bath and re-frozen at -80°C within 20 minutes after aliquotation. The study protocol was approved by the regional Ethical Review Agency in Stockholm, Sweden (Dnr 2013-2143-31-2). Informed written consent was obtained from all participants in accordance with the Declaration of Helsinki.

### Plasma analyses

NE was measured using the PMN Elastase Human ELISA Kit (Abcam, Cambridge, MA, USA, Cat# ab119553) and cfDNA using the Quant-iT PicoGreen dsDNA assay (Invitrogen, Carlsbad, CA, USA, Cat# P7589). These analyses were performed in EDTA plasma in the VEBIOS ER cohort and in citrated plasma in the DFW-VTE cohort. H3Cit-DNA was quantified in citrated plasma using an in-house capture ELISA as previously described [28] in both cohorts. D-dimer levels were measured using TinaQuant FEU (Roche, Basel, Switzerland) in citrated plasma in both cohorts. CRP levels were obtained from EMR. In DFW-VTE, D-dimer data was available for all patients as a result of it being one of the primary endpoints for the study. In VEBIOS ER, D-dimer was only available in patients where testing was considered clinically motivated, i.e. patients with low PTP.

### Statistics

To test for normal distribution, Shapiro-Wilk normality test was used. Standard deviations (SD) are reported for parametric data, medians and interquartile ranges (IQR) for non-parametric data. The unpaired student t test was used for parametric data, and the Mann Whitney U test for non-parametric data. Categorical variables are presented as proportions and compared using Fisher’s exact test. Discriminatory accuracy of the biomarkers, alone and in combination, for VTE was assessed using logistic regression analysis and presented as Area Under the Receiver Operating Characteristics curve (AUC). AUCs were compared using the Stata command roccomp. Complete case analysis was used and outliers were not excluded. Statistical analyses were performed using GraphPad Prism Software (GraphPad Prism 8, GraphPad Software, Inc., La Jolla, CA, USA) and R version 4.0.3. ROC curves for the different biomarker-based risk models were compared using the function roc.test in the RStudio attachment. All tests were two-tailed and a *P*-value < 0.05 was considered statistically significant.

## Results

### Patient characteristics

A total of 147 patients were included from each of the cohorts. VTE was confirmed in 51 patients in the VEBIOS ER cohort and in 61 in the DFW-VTE cohort. There were no significant differences in age or sex between VTE positive and VTE negative individuals within either of the cohorts. Among the healthy controls, the median age was 60 (30–69) years and 53% were males. The most common type of VTE was PE (61%) in the VEBIOS ER cohort and DVT (74%) in the DFW-VTE cohort. VTE positive patients in the DFW-VTE cohort more frequently had a history of VTE as well as recent trauma or surgery (p = 0.008 and p = 0.002 respectively). In both cohorts, increased plasma levels of CRP and D-dimer were associated with a confirmed VTE diagnosis (p < 0.0001). Demographic, clinical, and laboratory data are presented in Table 1.

**Table 1:** Demographic data, clinical characteristics and routine laboratory results of study participants.

	VEBIOS ER Cohort		DFW-VTE Cohort	
	VTE positive	VTE negative	VTE positive	VTE negative
	(n = 51)	(n = 96)	(n = 61)	(n = 86)
Age, median (IQR), years	57 (48–74)	61 (44–70)	67 (52–75)	68 (49–77)
Male sex, no. (%)	27 (53)	44 (46)	33 (54)	44 (51)
**VTE type**				
DVT confirmed, no. (%)	20 (39)		45 (74)	
Proximal, no. (%)	17 (85)		26 (58)	
Distal, no. (%)	3 (15)		19 (42)	
PE confirmed, no. (%)	31 (61)		16 (26)	
**VTE risk factors**				
Previous VTE, no. (%)	14 (27)	18 (19)	20 (33)	12 (14)
Active cancer, no. (%)	5 (9.8)	12 (12.5)	6 (9.8)	8 (9.3)
Trauma or surgery, no. (%)	3 (5.9)	4 (4.2)	7 (11)	0 (0)
Pregnancy, no. (%)	0 (0)	0 (0)	2 (3.3)	2 (2.3)
**Medications**				
Platelet inhibitors, no. (%)	3 (5.9)	16 (17)	8 (13)	20 (23)
Estrogen therapy, no. (%)	5 (9.8)	7 (7.3)	3 (4.9)	0 (0)
**Laboratory results**				
CRP, median (IQR), mg/L	17 (8–58)	5 (2–18)	12 (6–52)	4 (1–10)
D-dimer, median (IQR), mg/L FEU	1.7 (1.3–6.4)	0.52 (0.49–0.93)	3.4 (1.7–13)	0.46 (0.26–0.84)

VTE, venous thromboembolism; DVT, deep vein thrombosis; PE, pulmonary embolism; CRP, C-reactive protein; H3Cit-DNA; citrullinated histone H3; NE, neutrophil elastase; cfDNA, cell free DNA; IQR, interquartile range.

### Plasma levels of investigated markers and D-dimer

Plasma levels of investigated markers are presented in Table 2 and information on missing data can be found in S1 Table.

**Table 2:** Plasma concentration of markers of neutrophil activation and NETs.

	VEBIOS ER		DFW-VTE		Healthy
Biomarker	VTE positive	VTE negative	VTE positive	VTE negative	
H3Cit-DNA, median (IQR), ng/ml	110 (61–174)*	73 (32–147)	102 (46–173)**	54 (21–99)	38 (15–88)
NE, median (IQR), ng/ml	31 (24–40)**	24 (20–35)	49 (35–85)**	38 (23–54)	21 (16–27)
cfDNA, median (IQR), ng/ml	423 (377–478)	405 (361–461)	396 (353–459)	392 (350–466)	421 (396–445)

VTE, venous thromboembolism; H3Cit-DNA; citrullinated histone H3; NE, neutrophil elastase; cfDNA, cell free DNA; IQR, interquartile range. NS P > 0.05

* P < 0.05

** P < 0.01

***P < 0.001

**** P < 0.0001.

#### Circulating H3Cit-DNA and NE were elevated in patients with VTE

**Fig 2:**
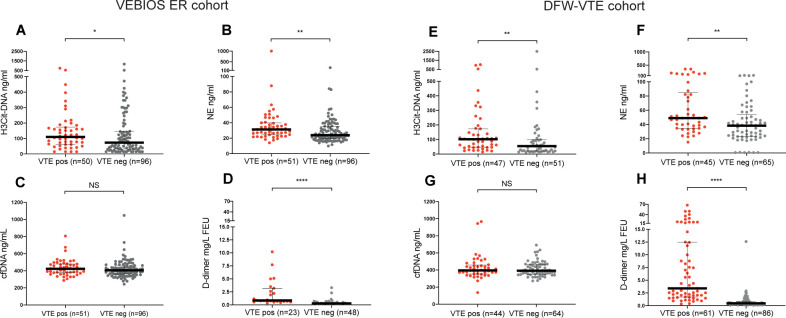
Plasma levels of investigated markers. Increased plasma levels of H3Cit-DNA and NE but not cfDNA was observed in VTE positive study particants compared to VTE negative study participants in both the VEBIOS ER cohort (A-C) and the DFW-VTE cohort (D-F). In the healthy control group (G-I), H3Cit-DNA and NE levels were generally lower compared to the VTE cohorts. However, no difference in plasma levels of cfDNA was observed when compared to VTE negative or VTE positive participants. Lines represent medians with IQR. NS P > 0.05, * P < 0.05, ** P < 0.01, ***P < 0.001, **** P < 0.0001.

H3Cit-DNA was elevated in VTE positive cases compared to VTE negative cases in both the VEBIOS ER cohort (p = 0.040, Fig 2A) and the DFW-VTE cohort (p = 0.003, Fig 2D). Median levels in VTE cases were similar in both cohorts (p = 0.51). A similar difference was observed for NE in both the VEBIOS ER cohort (p = 0.002, Fig 2B) and the DFW-VTE cohort (p = 0.001, Fig 2E). Median concentrations of NE were generally higher in the DFW-VTE cohort, both when comparing VTE positive cases (p<0.0001) and VTE negative cases (p = 0.0004) than in the VEBIOS ER cohort. In both cohorts, H3Cit-DNA levels were higher in VTE negative cases compared to healthy controls (Fig 2G), however only reaching significance in VEBIOS ER (p = 0.033). For NE (Fig 2H), a similar pattern was observed with higher levels in VTE negative patients in both VEBIOS ER (p = 0.014) and DFW-VTE (p<0.0001).

#### No association between plasma levels of cfDNA and VTE

Notably, there were no differences in the levels of plasma cfDNA between VTE positive and VTE negative cases in any of the cohorts (p = 0.28 for VEBIOS ER (Fig 2C) and p = 0.71 for DFW-VTE (Fig 2F). Further, no difference in the levels of plasma cfDNA was observed between the two cohorts (VTE positive p = 0.34; VTE negative p = 0.60). To allow comparison with prior studies, subgroup analyses were performed within each cohort. However, this did not change the results showing no differences between PE positive and PE negative cases (VEBIOS ER p = 0.37; DFW-VTE p = 0.90) or DVT positive and DVT negative cases (VEBIOS ER p = 0.99; DFW-VTE p = 0.75). Finally, no significant differences regarding cfDNA levels in healthy controls were observed when compared to the VTE cohorts, regardless of VTE status.

#### D-dimer was strongly associated with VTE

D-dimer was associated with VTE in both cohorts (p<0.0001). A difference was observed between the cohorts amongst VTE negative patients (p = 0.0007) with higher levels in the VEBIOS ER cohort. An opposite trend was observed in VTE positive cases with higher levels in the DFW-VTE cohort but without reaching statistical significance (p = 0.05). Using age-adjusted cut-offs [10] we calculated study specific sensitivity, 1.00 and 0.97, and specificity, 0.60 and 0.67, for VTE in VEBIOS ER and DFW-VTE respectively. These findings are in line with the results from prior studies in similar settings [29].

#### The predictive performance of D-dimer alone is superior to risk models based on H3Cit-DNA and/or NE

In both cohorts, risk models consisting of H3Cit-DNA, NE and D-dimer were all able to discriminate VTE positive from VTE negative cases (Table 3). Upon comparison, the D-dimer based model was superior to both NE (p = 0.008 and p<0.0001 in VEBIOS ER and DFW-VTE, respectively) and H3Cit-DNA (p<0.0001 in VEBIOS ER and DFW-VTE, respectively). A risk model based on both NE and H3Cit-DNA was still inferior to D-dimer alone (p = 0.003 and p = 0.004 for VEBIOS ER and DFW-VTE respectively). Finally, combining D-dimer with NE and/or H3Cit-DNA did not further increase the accuracy to predict VTE.

**Table 3:** Area under receiver operating characteristics curve (AUC) for D-dimer, H3Cit-DNA and NE.

	VEBIOS ER		DFW-VTE	
Blood biomarker	n	AUC (95% CI)	n	AUC (95% CI)
D-Dimer	71	0.90 (0.84–0.97)	147	0.93 (0.89–0.97)
NE	147	0.65 (0.56–0.74)	110	0.68 (0.58–0.78)
H3Cit-DNA	146	0.60 (0.51–0.70)	98	0.67 (0.57–0.78)
H3Cit-DNA + NE	146	0.65 (0.54–0.73)	61	0.76 (0.64–0.88)
H3Cit-DNA + D-dimer	71	0.90 (0.84–0.97)	98	0.93 (0.89–0.98)
NE + D-dimer	71	0.90 (0.84–0.97)	110	0.92 (0.87–0.97)
H3Cit-DNA + NE + D-dimer	71	0.90 (0.84–0.97)	61	0.94 (0.88–0.99)

CI; confidence interval; H3Cit-DNA, citrullinated histone H3 DNA; NE, neutrophil elastase

We also performed subgroup analysis, evaluating the discriminatory accuracy of NE and H3Cit-DNA based risk models in patients with a positive age-adjusted, D-dimer test (S2 Table). A combination of H3Cit-DNA and NE was able to distinguish VTE positive from VTE negative cases in both cohorts as opposed to H3Cit-DNA alone. The results for NE was inconsistent between the cohorts.

Finally, predictive performance of NE and H3Cit-DNA in combination with D-dimer was also evaluated by difference in continuous net reclassification improvement (cNRI) and integrated discrimination improvement (IDI) compared to a base model of D-dimer alone (S3 Table). These analyses did not indicate any added value of NE or H3Cit-DNA to D-dimer alone.

## Discussion

This is the first study demonstrating an association between plasma levels of NET specific marker H3Cit-DNA and VTE. In addition, increased levels of the neutrophil activation marker NE were also observed in VTE positive cases. These findings are in line with prior data on the role of NET formation in VTE. However, neither H3Cit-DNA nor NE provided any added value to D-dimer in assessing the clinical probability of VTE suggesting a limited role as diagnostic biomarkers.

Prior studies investigating the role of NETs in clinical VTE diagnostics have employed surrogate NET markers, such as cfDNA, nucleosomes and MPO [21, 23]. There are five studies showing elevations of cfDNA in PE patients [30–34] and three studies showing no elevations in this patient population [35–37]. More recently, granular proteins have been examined as potential diagnostic markers in DVT patients. van Montfoort et al. assessed plasma levels of circulating nucleosomes and NE in 345 patients; 150 patients with confirmed symptomatic DVT and 195 patients with a clinical suspicion of DVT but in whom DVT was excluded [23]. High levels of both markers were associated with an increased probability of DVT. Similar results were obtained in another study investigating cfDNA and MPO in 47 patients with DVT compared to 28 patients with a clinical suspicion of DVT but in whom DVT was excluded [21]. A study investigating a biomarker panel of fifty diagnostic candidates in PE revealed a high discriminatory performance of MPO, only outperformed by D-dimer [38]. Although some of these markers are clearly associated with VTE, their clinical utility remains unclear. We found no elevations of cfDNA in VTE patients in either of the cohorts used in this study.

Notably, elevations of circulating H3Cit have been observed in several conditions associated with an increased risk of VTE, including infection [39], cancer [40], autoimmune disease [41] and arterial thrombosis [42]. The increased plasma levels of H3Cit-DNA and NE observed in VTE patients in both of our study cohorts could therefore also be associated with the underlying cause of VTE in addition to be a consequence of VTE itself. We believe that these circumstances are reflected in the limited diagnostic value of the investigated markers as indicated by the AUC when compared to D-dimer.

In theory, novel markers with low correlation to D-dimer but nonetheless strong association to VTE have a better potential to complement the current diagnostic strategy. From a pathophysiological perspective, tentative markers include those reflecting activation and/or dysfunction of endothelial cells and platelets, hypercoagulation as well as thrombus formation and stabilization, rather than fibrinolysis. Historically, markers of inflammation, for example CRP and IL-6, have been evaluated and associated with occurrence and extent of VTE [43–45]. However, in a clinical context distinguishing thromboinflammation from other inflammatory processes may be a challenging task and this perhaps explains why none of the proposed markers have reached clinical implementation. Thanks to omics-based research, a potential way to address the often complex etiology of VTE, for example including both infection and inflammation, is to use a panel of proteins in combination with clinical risk factors to improve diagnostics.

Savchenko et al. [19] found that H3Cit and other NET components predominantly were localized in organizing venous thrombi, as opposed to unorganized and organized thrombi. Similarly, De Boer et al. [46] identified the presence of NETs in fresh but not older coronary artery thrombi. Taken together, these findings could suggest that NETs primarily play a role in early thrombus formation and possibly degrades over time. Therefore, it is reasonable to assume that thrombus age also affects the plasma concentration of circulating NETs components. Even though all research subjects were sampled upon ER admission, the time from clot formation and symptom onset to blood draw can be assumed to vary considerably. With stricter inclusion criteria in regard to time from symptom onset to sample collection it is possible that the investigated markers would have shown a stronger association and higher specificity to VTE. This could perhaps therefor be considered a limitation in our study. However, we do not believe it has serious effects on the possibility to answer the main question of the study, i.e. the potential role of NET markers in VTE diagnostics. Biomarkers with very specific requirements in regard to duration of symptoms and preanalytical handling have a much smaller chance to reach clinical implementation.

There are also some other limitations to consider in this study, for example the post-hoc design, as well as a varying degree of missing laboratory data between the cohorts. We acknowledge a systematic difference in NE levels between the cohorts and attribute this to differences in preanalytical handling and preparation of the plasma.

This study is strengthened by the consistency of results across two large, independent cohorts of VTE patients, indicating a high generalizability and external validity. Furthermore, both cohorts comprise of both PE and DVT patients. The inclusion of patients prior to establishing VTE diagnosis and initiation of anticoagulant treatment, as well as the control group of patients with initially suspected VTE where the diagnosis was ruled out, further strengthens the result. The results were consistent across cohorts despite a higher rate of DVT in DFW-VTE, supporting our decision to analyze VTE positive patients on an aggregated level instead of subcategorized into PE and DVT.

## Conclusions

Neutrophil activation marker NE and NET specific marker H3Cit-DNA are elevated in patients with acute VTE. However, the diagnostic accuracy of these markers does not exceed that of the clinically used D-dimer. Furthermore, the addition of NE or H3Cit-DNA to D-dimer did not improve the diagnostic accuracy of D-dimer, suggesting a limited use in a clinical VTE setting.

## Supporting information

S1 Table Missing data for investigated markers and D-dimer. (DOCX)

S2 Table Predictive performance for H3Cit-DNA and NE in D-dimer positive subgroups in each cohort. (DOCX)

S3 Table Predictive performance for H3Cit-DNA and NE in combination with D-dimer compared to a base model of D-dimer alone by difference in continuous net reclassification improvement (cNRI) and integrated discrimination improvement (IDI) analysis. (DOCX)
