# Supplementary material for: Markers of neutrophil activation and neutrophil extracellular traps in diagnosing patients with acute venous thromboembolism: A feasibility study based on two VTE cohorts
Source: PLoS One. 2022 Jul 28;17(7):e0270865. doi: 10.1371/journal.pone.0270865 (PMC9333265; doi:10.1371/journal.pone.0270865)
Supplement: S1 Table — (DOCX) [file pone.0270865.s001.docx]

**S1 Table**. Missing data for investigated markers and D-dimer.

|  | **VEBIOS ER** | | **DFW-VTE** | | **Healthy** |
| --- | --- | --- | --- | --- | --- |
| **Biomarker** | VTE positive (n=51) | VTE negative (n=96) | VTE positive (n=61) | VTE negative (n=86) | (n=30) |
| H3Cit-DNA, no. (%) | 1 (2) | 0 | 14 (23) | 35 (41) | 2 (7) |
| NE, no. (%) | 0 | 0 | 16 (26) | 21 (24) | 0 |
| cfDNA, no. (%) | 0 | 0 | 17 (28) | 22 (26) | 0 |
| D-dimer, no. (%) | 28 (55) | 48 (50) | 0 (0) | 0 (0) | 30 (100) |
