# Supplementary material for: Markers of neutrophil activation and neutrophil extracellular traps in diagnosing patients with acute venous thromboembolism: A feasibility study based on two VTE cohorts
Source: PLoS One. 2022 Jul 28;17(7):e0270865. doi: 10.1371/journal.pone.0270865 (PMC9333265; doi:10.1371/journal.pone.0270865)
Supplement: S2 Table — (DOCX) [file pone.0270865.s002.docx]

**S2 Table**. Predictive performance for H3Cit-DNA and NE in D-dimer positive subgroups in each cohort.

|  | **VEBIOS ER (n=42)** | | **DFW-VTE (n=87)** | |
| --- | --- | --- | --- | --- |
| **Biomarker** | **n** | **AUC** | **n** | **AUC** |
| H3Cit-DNA | 42 | 0.49 (0.31-0.68) | 59 | 0.48 (0.28-0.68) |
| NE | 42 | 0.70 (0.53-0.87) | 68 | 0.61 (0.47-0.75) |
| H3Cit-DNA + NE | 42 | 0.70 (0.53-0.87) | 40 | 0.72 (0.55-0.90) |
