# Supplementary material for: Markers of neutrophil activation and neutrophil extracellular traps in diagnosing patients with acute venous thromboembolism: A feasibility study based on two VTE cohorts
Source: PLoS One. 2022 Jul 28;17(7):e0270865. doi: 10.1371/journal.pone.0270865 (PMC9333265; doi:10.1371/journal.pone.0270865)
Supplement: S3 Table — (DOCX) [file pone.0270865.s003.docx]

**S3 Table**. Predictive performance for H3Cit-DNA and NE in combination with D-dimer compared to a base model of D-dimer alone by difference in continuous net reclassification improvement (cNRI) and integrated discrimination improvement (IDI) analysis.

| **Cohort/risk model** | **n** | **cNRI (95% CI)** | **IDI (95% CI)** |
| --- | --- | --- | --- |
| DFW-VTE |  |  |  |
| H3Cit-DNA + DDI | 98 | 0.014 [-0.324 - 0.351]; p=0.936 | 0.004 [-0.005 - 0.012]; p=0.404 |
| NE + DDI | 110 | -0.063 [-0.414 - 0.287]; p=0.724 | 0.001 [-0.017 - 0.019]; p=0.875 |
| VEBIOS ER |  |  |  |
| H3Cit-DNA + DDI | 71 | -0.290 [-0.778 - 0.198]; p=0.244 | 0.0001 [-0.001 - 0.001]; p=0.885 |
| NE + DDI | 71 | 0.226 [-0.152 - 0.605]; p=0.241 | 0.029 [-0.030 - 0.087]; p=0.334 |
